# Case report: Omphalitis caused by *Trueperella pyogenes* infection in a Korean indigenous calf

**DOI:** 10.3389/fvets.2024.1362352

**Published:** 2024-05-30

**Authors:** Youngjun Kim, Min-Jeong Ji, Jinho Park, Kyoung-Seong Choi

**Affiliations:** ^1^Department of Animal Hospital, Hanwoo (Korean Indigenous Cattle) Genetic Improvement Center, National Agricultural Cooperative Federation, Seosan, Republic of Korea; ^2^Department of Veterinary Internal Medicine, College of Veterinary Medicine, Jeonbuk National University, Iksan, Republic of Korea; ^3^Department of Animal Science and Biotechnology, College of Ecology and Environmental Science, Kyungpook National University, Sangju, Republic of Korea

**Keywords:** *Trueperella pyogenes*, omphalitis, failure of passive transfer, antimicrobial susceptibility patterns, Korean indigenous calf

## Abstract

Omphalitis, commonly caused by opportunistic bacteria has been significantly associated with morbidity and mortality in neonatal calves. *Trueperella pyogenes* is a commensal and opportunistic pathogen that can cause suppurative infection in farm animals. Our case involved a 10-day-old female Korean indigenous calf that presented with umbilical enlargement accompanied by a greenish-yellow purulent discharge and right forelimb lameness. The calf was diagnosed with failure of passive transfer at 24 h of age. Physical examination found hypothermia (38.1°C), tachycardia (110 beats/min), tachypnea (47 cycles/min), and open mouth breathing. Ultrasonography revealed hyperechoic pus in the 9th and 10th right intercostals, for which a liver abscess due to omphalophlebitis was suspected. After 3 days, the calf died. *T. pyogenes* was detected in the umbilical cord, lung, liver, kidney, intestine, mesenteric lymph node, urinary bladder, and bladder ligament. All genes related to the virulent factors (i.e., *plo*, *cbpA, fimA, fimC, fimG, nanH,* and *nanP*) were also identified, with *plo* and *fimA* being associated with pathogenicity. A final diagnosis of omphalitis was established based on the identification of virulent *T. pyogenes* and umbilical cord dilatation on ultrasonography. Antimicrobial susceptibility tests showed that the isolated *T. pyogenes* was susceptible to amoxicillin, ceftiofur, florfenicol, enrofloxacin, ofloxacin, and ciprofloxacin, suggesting the suitability of these antibiotics for treating *T. pyogenes*-induced omphalitis. Hence, accurate and rapid diagnosis of the involved bacteria and antimicrobial susceptibility patterns can help guide therapeutic decisions. Our case provides useful information that could aid large animal clinicians in the diagnosis and treatment of *T. pyogenes*-induced omphalitis.

## Introduction

Omphalitis, often referred to as “navel ill” in farm animals, is an inflammation of the umbilicus and its connective tissue that mainly occurs on the extra-abdominal portion of the umbilicus approximately the first 30 days of life in calves. Although this condition is quite common among neonatal calves, no report has detailed its incidence aside from those that reported morbidity rates of 1.3 and 29.9%. Cleanliness of the calving and calf housing areas, transfer of passive immunity, and umbilical cord management of newborn calves are known to be associated with the occurrence of omphalitis. Various bacteria, such as members of family *Enterobactericeae* and Gram-positive cocci, have also been reported to cause omphalitis (1–3). Hence, the identification of the causative organism may be necessary for effective treatment.

*Trueperella pyogenes* is a Gram-positive, pleomorphic, non-spore-forming, non-motile, and facultatively anaerobic bacterium that has been identified as a commensal and opportunistic pathogen found in the mucous membranes of the upper respiratory, gastrointestinal, and urogenital tracts of farm animals (4–6). *T. pyogenes* is a major causative agent of mastitis, metritis, liver abscesses, lymphadenitis, otitis, peritonitis, pneumonia, pyodermitis, osteomyelitis, arthritis, endocarditis, umbilical thickening, and septicemia (4, 5, 7–9). *T. pyogenes* infections have been associated with decreased milk production and low quality of meat products, leading to severe economic losses in the livestock industry (10). The pathogenicity of *T. pyogenes* can be attributed to several virulence factors. Among them, pyolysin (*plo*), which is associated with tissue damage, has been considered the primary virulence factor of this pathogen, along with neuraminidases (*nanH* and *nanP*), fimbriae (*fimA, fimC, fimE,* and *fimG*), and collagen-binding protein (*cbpA*), which have been associated with virulence, mucosal adherence, and colonization (5). Sporadic cases of *T. pyogenes* infections that result in endocarditis and soft tissue infections have also been reported in humans (11, 12).

The umbilical cord is an organ that transmits nutrients and oxygen between the mother and her fetus (13). Following parturition, the umbilical cord of the calf that is cut and left behind dries and detaches, leaving a structure known as the navel (13). During this drying period, the umbilical cord is exposed to a contaminated environment that allows pathogens to easily enter the umbilical cord, causing infection of the urachus, umbilical arteries (omphaloarteritis), or umbilical vein (omphalophlebitis) (13). An infection in any or all umbilical structures (omphalitis) manifests as signs of inflammation, such as localized heat, swelling, purulent discharge, and pain, which may cause systemic infections, such as arthritis, peritonitis, pneumonia, uveitis, and even sepsis, due to the hematogenous spread of bacteria ascending along the structures of the umbilical cord (3).

Omphalitis is the third most common disease in newborn calves following neonatal diarrhea and bovine respiratory disease (BRD). Nevertheless, the available information regarding this condition in cattle is limited (14). We herein report a case of omphalitis caused by *T. pyogenes*, with subsequent pneumonia, arthritis, and liver abscess, in a Korean native calf, as well as present appropriate guidelines for its treatment. This case provides useful information that could help large animal clinicians diagnose and treat omphalitis, which is commonly observed on farms.

## Case presentation

A 10-day-old female Hanwoo calf (*Bos taurus coreanae*) presented to our clinic with an enlarged umbilicus and right forelimb lameness as the main complaints. Failure of passive transfer (FPT) was diagnosed when the calf was 24 h of age, with a serum biochemical profile showing a total protein of 4.1 g/dL and globulin of 1.5 g/dL. The calf was also mildly anemic, as indicated by a hematocrit of 23.1% (Supplementary Figure S1). At the time of FPT diagnosis, the dam was not managing the calf, which did not exhibit any suckling reflex. A blood transfusion was performed at a dose of 15 mL/kg to treat the FPT, and the calf was subsequently reared on artificial milk. After the transfusion, the total protein increased slightly to 5.1 g/dL, but the calf was still weak and had no suckling reflex. Thus, it was force-fed 1,000–1,500 mL/day of replacement milk.

**Figure 1:**
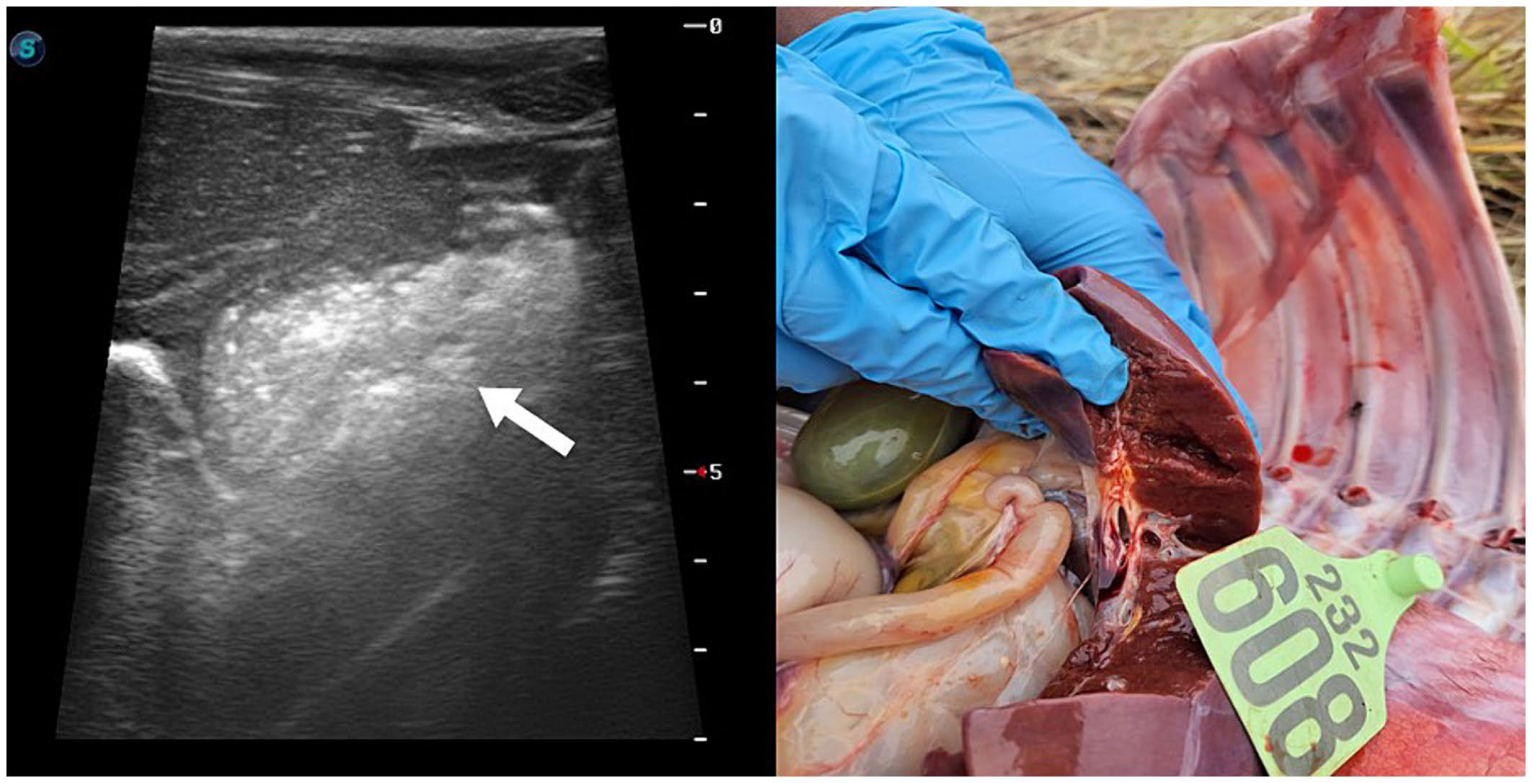
Ultrasonography images showing a hyperechoic lesion at adjacent to the liver and umbilical vein in the 10-day-old female Hanwoo calf.

Upon arrival at the clinic, the calf had a temperature of 38.1°C, indicating mild hypothermia, a heart rate of 110 beats/min with tachycardia, and a respiratory rate of 47 cycles/min with tachypnea and open mouth breathing. Auscultation of the chest revealed crackles in the anterior lobe of the right lung. Swelling was observed at the knee of the right forelimb, which was warm to the touch. In addition, purulent material was noted on the umbilicus, which was warm but not painful. Ultrasonography revealed hyperechoic pus in the right 9th and 10th intercostals, adjacent to the liver and umbilical vein (Figure 1), as well as bladder distention. Based on the examination findings, a liver abscess due to omphalophlebitis was suspected, for which surgery was indicated. However, the calf’s condition failed to stabilize. After 3 days of treatment with penicillin at 22,000 IU/kg, surgical repair was planned. Unfortunately, the calf died within 2 h of diagnosis.

**Figure 2:**
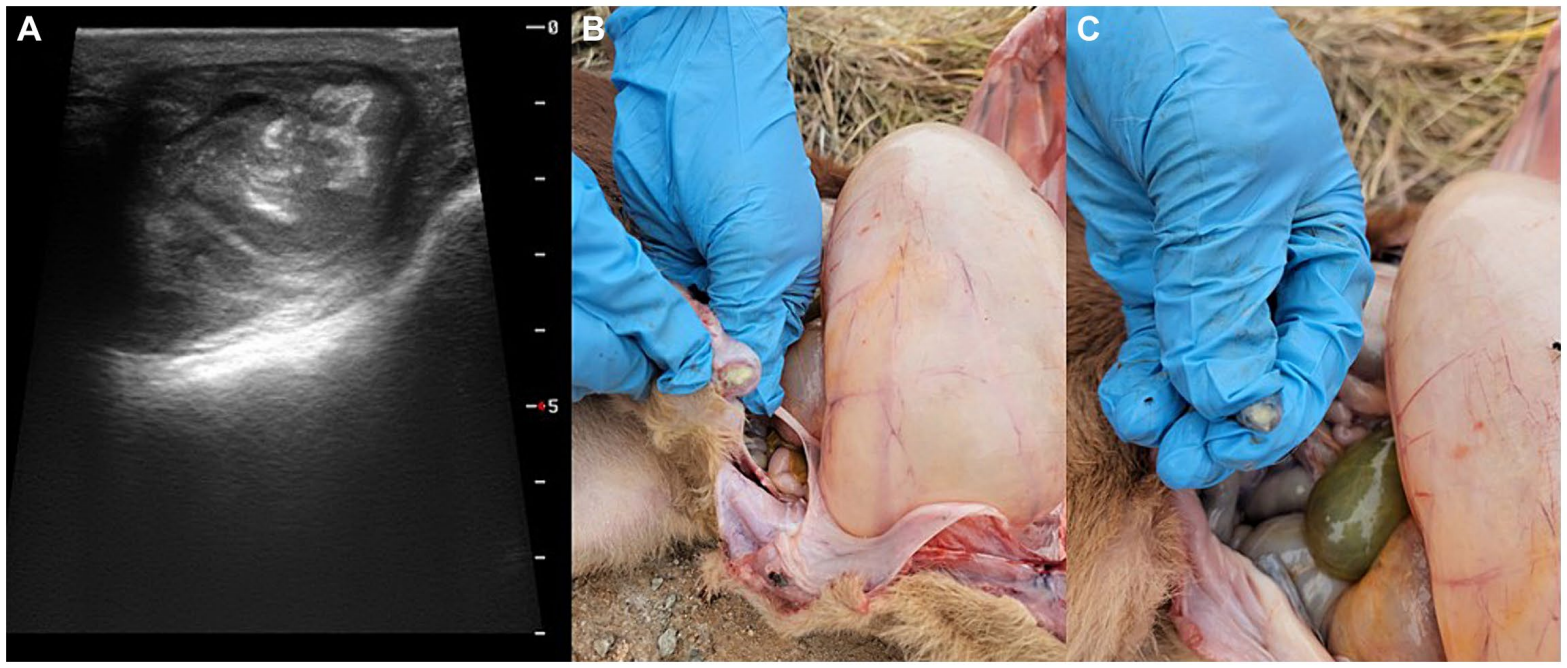
Gross lesions of the umbilical cord and bladder ligaments vein in the 10-day-old female Hanwoo calf presenting **(A)** umbilical enlargement, **(B)** umbilical vein, and **(C)** umbilical artery with purulent discharge.

**Figure 3:**
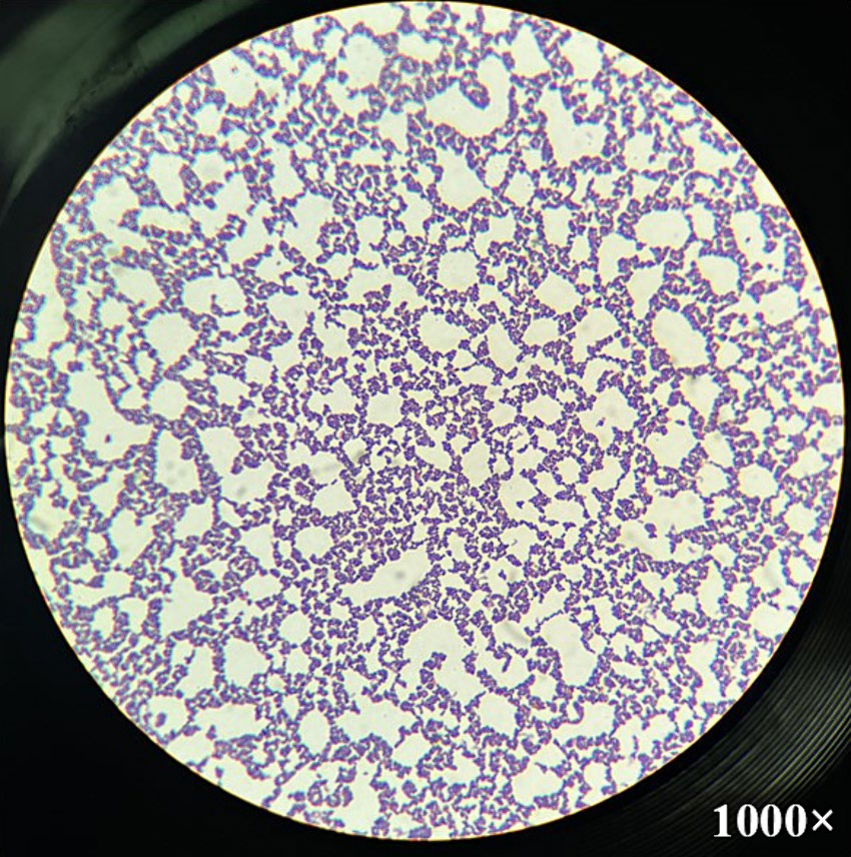
Microscopic image of *Trueperella pyogenes* after Gram-staining. Gram-positive coccobacilli were observed from the single colony cultured in greenish-yellow purulent discharge from the umbilical cord at magnification 1,000×.

At necropsy, the umbilical cord and bladder ligament, where purulent substances were observed, were collected and transported to the laboratory (Figure 2). After the umbilical cord was incised, a greenish-yellow substance was found on the inner surface. The internal suppurative material was cultured on blood agar plates for 48 h at 37°C under facultative anaerobic condition. The colony was white, smooth, and glossy and surrounded by a narrow zone of hemolysis. Morphologically, distinct colonies were selected, and Gram-staining was performed, which confirmed the presence of Gram-positive bacteria (Figure 3). The single colony of Gram-positive bacteria was inoculated into brain heart infusion (BHI) broth and incubated overnight at 37°C. The bacterial DNA was extracted using the QIAamp UCP Pathogen Mini Kit (Qiagen, Hilden, Germany) according to the manufacturer’s instructions. *T. pyogenes* isolate (provided by Yong-Il Cho, Sunchon National University, Sunchon, ROK) was cultivated at 37°C in BHI broth and used as positive control (15).

**Figure 4:**
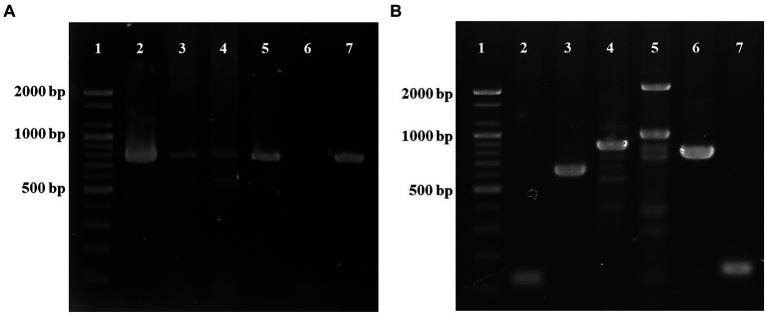
PCR analysis for the detection of *Trueperella pyogenes* using the *plo* gene (773 bp) **(A)**. Lane 1: ladder (1 kb), lane 2: umbilical cord, lane 3: lung, lane 4: mesenteric lymph nodes, lane 5: bladder, lane 6: negative control, lane 7: positive control. PCR analysis of virulent genes **(B)**. Lane 1: ladder (1 kb), lane 2: collagen-binding protein (*cbpA*, 124 bp), lane 3: type A fimbriae (*fimA,* 605 bp), lane 4: type C fimbriae (*fimC*, 843 bp), lane 5: type G fimbriae (*fimG*, 929 bp), lane 6: neuraminidase H (*nanH*, 781 bp), lane 7: neuraminidase P (*nanP*, 150 bp).

DNA was extracted from the umbilical cord, umbilical vein, umbilical artery, lungs, kidneys, intestines, mesenteric lymph nodes, and bladder using the DNeasy Blood & Tissue Kit (Qiagen) according to the manufacturer’s instructions. Polymerase chain reaction (PCR) analysis was conducted to detect the genes of *T. pyogenes* infection. For detection of the *plo* gene, a specific primer set (F: 5′-CAGTCAAGGTGAGTGAGTGGAAA-3′ and R: 5′-CTTGAACTGGGAAA-3′) was used (16). The predicted sized of the amplicon was 773 bp using the following cycling conditions: 94°C for 5 min, followed by 40 cycles at 94°C for 30 s, 47°C for 30 s, and 72°C for 50 s. Distilled water was included in each PCR run as a negative control. *T. pyogenes* was detected in all the samples examined (Figure 4A). *Escherichia coli* was also tested from all the tissue samples (17), and the hemolysin gene was found only in the kidney and intestine.

Additionally, as gross lesions were markedly found in the lungs, PCR analysis was also performed to detect the main pathogens causing BRD, such as *Mannheimia haemolytica, Pasteurella multocida, Histophilus somni, E. coli*, and *T. pyogenes*. However, no other bacteria besides *T. pyogenes* were detected in the lungs. All PCR products were separated by electrophoresis on 1.2% agarose gels and visualized after staining with ethidium bromide. The *T. pyogenes*-positive PCR products were purified using the Accupower PCR Purification Kit (Bioneer, Daejeon, ROK) according to the manufacturer’s instructions and directly sequenced (Bioneer). The nucleotide sequence obtained in this study was aligned using the BioEdit software and then compared with reference sequences from the National Center for Biotechnology Information database^1^ to determine its similarity using Geneious Prime 2022.2 software.^2^ Phylogenetic analysis of *T. pyogenes* was performed using the maximum-likelihood method implemented in MEGA11 using the best substitution model. Bootstrap values were calculated by analyzing 1,000 replicates to evaluate the reliability of clusters, and the sequence obtained in this calf was grouped with the reference strains of *T. pyogenes* (Supplementary Figure S2). Our sequence showed 97.3–99.1% identity to those detected in other countries. The nucleotide sequence obtained in this study was assigned accession number PP731570.

Subsequently, the presence of virulence genes, including *cbpA, fimC, fimA, fimG*, *nanP, nanH,* and *plo*, was investigated in *T. pyogenes*-positive isolates using specific primers (Supplementary Table S1), and all the virulence genes were identified (Figure 4B).

Antimicrobial susceptibility tests of the isolated *T. pyogenes* were conducted using the disk diffusion method on Mueller-Hilton agar with the following 12 antibacterial disks: amoxicillin (10 μg), penicillin G (10 IU), ceftiofur (30 μg), oxytetracycline (30 μg), florfenicol (30 μg), enrofloxacin (5 μg), ofloxacin (5 μg), ciprofloxacin (5 μg), erythromycin (15 μg), streptomycin (10 μg), lincomycin (2 μg), and clindamycin (2 μg). The results of antimicrobial susceptibility tests were recorded after 48 h of incubation. For amoxicillin, penicillin G, ceftiofur, florfenicol, enrofloxacin, ofloxacin, ciprofloxacin, erythromycin, lincomycin, and clindamycin, criteria for *T. pyogenes* were used to interpret the results and catalog the strains as susceptible or non-susceptible according to CLSI (2023). For the rest of antimicrobial agents, no standards are available. Accordingly, the *T. pyogenes* isolates obtained from omphalitis were found to be susceptible to amoxicillin, ceftiofur, florfenicol, enrofloxacin, ofloxacin, and ciprofloxacin but showed high resistance to the remaining antibiotics (Supplementary Table S2).

## Discussion

In newborn calves, the umbilicus, along with the gastrointestinal and respiratory tracts can serve as entry points for infection. Several pathogens cause infectious neonatal diseases by exploiting the umbilicus as the main route of entry. Omphalitis contributes significantly to neonatal morbidity in cattle (18, 19). Previous studies have shown that umbilical infections occur in 1.3 to 14% of dairy calves (20, 21). According to a recent study, omphalitis is the most commonly detected disorder in pre-weaned calves, with the largest proportion being 15.9% at herd level (22). Additionally, omphalitis has been found to cause 23% of preslaughter mortality and 54% of post-slaughter in veal calves (23). Although omphalitis can cause growth retardation, increased susceptibility to other diseases, and higher mortality rates, this has not garnered much attention from farmers and veterinarians.

Good sanitation at parturition, maternity pen hygiene, cleanliness of the calf environment, proper colostrum intake, and umbilical disinfection have been reported as management practices that reduce the incidence of omphalitis in newborn calves (24). In the current case, the calf was diagnosed with FPT, with a total serum protein of 4.1 g/dL. Despite receiving blood transfusion, this calf still exhibited signs of FPT and no suckling reflex. The calf was not born in dystocia, had a well-maintained and hygienic maternity pen, and did not spend much time in the cow-calf barn due to artificial lactation. Although FPT was considered to cause omphalitis, recent studies have reported that FPT does not cause omphalitis (14). However, the occurrence of omphalitis in a calf with FPT, as in the current case, is believed to increase septicemia through hematogenous transmission.

The distinction between local and systemic omphalitis infection in newborn calves is clinically important because these differences can affect both prognosis and treatment decisions. The diagnosis of omphalitis in neonatal calves is primarily based on palpation, ultrasonography, and aspiration (25–27). Palpation is probably the most frequently used approach in clinical practice given its simplicity and availability. In cases of omphalophlebitis or omphaloarteritis, which are usually not localized infections, deep palpation may reveal dilated veins or signs of pain in the calf. In this case, however, while the calf exhibited both omphalophlebitis and omphaloarteritis, it did not show any signs of pain on palpation. In this case, dilatation of the umbilical vein or arteries was not palpable on deep palpation and was not observed on ultrasonography. At necropsy, pus was found in the umbilical vein and arteries, but no dilatation could be seen in both. This may be explained by the result of chronic infection with omphalitis. The calf was primarily in sternal or lateral recumbency and was very depressed. As demonstrated in the current case, ultrasonography should be considered as an adjunct to visualization and palpation in septic calves. Indeed, studies have shown that ultrasonography can be used to determine the site and extent of the infection, as well as detect the dilatation of the umbilical veins and arteries with hypo/hyper echogenic fluid (26–29).

In the current case, the bacteria detected in the umbilical cords were identified as *T. pyogenes* by PCR method, and this was confirmed by sequencing analysis. However, a limitation of our study is that biochemical tests, including urease, oxidase, catalase, nitrate reduction, gelatin hydrolysis, and fermentation of sucrose, mannitol, maltose, lactose, glucose, and xylose, for identification of *T. pyogenes* were not performed. Nevertheless, the possibility of *T. pyogenes* as the causative organism can be considered by colony morphology and Gram-staining results. In general, the initial identification of bacteria is based on cell morphology and colony characteristics (6). The colonies cultured in the umbilical cords typically exhibited beta-hemolysis zones with hemolytic rings on blood agar and the glistering colonies were morphologically identified as *T. pyogenes*. In addition to morphological characteristics (30), the clinical presentation of the calf, particularly the presence of hyperechoic pus, pointed to a potential *T. pyogenes* infection. *T. pyogenes* is a common pathogen implicated in various clinical manifestations among farm animals (8, 31), which it is an important opportunistic pathogen, particularly associated with suppurative infections (4, 6, 8). Its prevalence has recently been increasing worldwide (32). However, reports of omphalitis caused by *T. pyogenes* in cattle have been very limited (8), with only one related case described in foals (3). The present study demonstrated that *T. pyogenes* can cause omphalitis. Currently, the transmission route of this bacteria remains unclear. However, we assumed that this calf became infected by contact between the umbilical cord and contaminated utensils or environment. Another possible route of entry for this pathogen could be the gastrointestinal tract, indicating that infection may be caused by dissemination of septicemia in the vessels.

Moreover, all genes for virulence factors were identified in this calf. Studies have been shown differences in the expressions of genes encoding virulence factors among various clinical manifestations (5). In particular, *plo* and *fimA* virulence factors have been identified as highly crucial to the pathogenesis of *T. pyogenes* infection and useful genetic markers in the detection of this pathogen in clinical samples (5, 33). Considering that other virulence genes aside from these two were expressed in the current case, the *T. pyogenes* detected in this study can be considered virulent. Although few reports have been available on *T. pyogenes* infections in humans, farm animals have been considered a major source of human infection. Hence, the risk to public health due to pathogenic *T. pyogenes* through animal-to-human transmission should not be overlooked.

The clinical course of these suppurative infections caused by *T. pyogenes* may be severe, with misdiagnosis or inappropriate treatment only leading to increased mortality rates (6). As such, bacterial identification and antimicrobial susceptibility tests should be performed to decide the appropriate treatment plan. Generally, cephalosporins, penicillin and other beta-lactams, tetracyclines, and macrolides are the first line of antimicrobial therapies of choice for *T. pyogenes* infections in livestock (2, 27, 34). However, the *T. pyogenes* isolates identified in the current case were found to be mainly resistant to penicillin G, oxytetracycline, erythromycin, streptomycin, lincomycin, and clindamycin but susceptible to amoxicillin, ceftiofur, florfenicol, enrofloxacin, ofloxacin, and ciprofloxacin. Moreover, previous studies have reported that *T. pyogenes* are resistant to macrolides, tetracyclines, and beta-lactam antibiotics (33, 35–37), which is somewhat consistent with our results. Although macrolides and penicillin had been frequently used to treat *T. pyogenes* infections in the past, the inappropriate use of antimicrobials increases the selection rate of multidrug-resistant bacteria. Especially in the current case, the resistance of *T. pyogenes* to penicillin G may be attributed to its inefficacy against slow-growing bacteria and inability to infiltrate biological membranes. Quinolones (e.g., enrofloxacin, ofloxacin, and ciprofloxacin) and amphenicols (e.g., florfenicol) primarily targeted Gram-negative bacteria and have been approved for the treatment of BRD in cattle. Interestingly, these antibiotics were found to be effective against *T. pyogenes* in this case. Therefore, the appropriate and responsible use of antimicrobials for farm animals can prevent the emergence of multidrug-resistant bacterial pathogens and mitigate one health concerns.

## Conclusion

This case describes the occurrence of omphalitis caused by *T. pyogenes* in a 10-day-old Hanwoo calf. All the virulence genes were detected in this case. Moreover, *T. pyogenes* isolated in this study are susceptible to amoxicillin, ceftiofur, florfenicol, enrofloxacin, ofloxacin, and ciprofloxacin, suggesting the use of these antibiotics as the first line of treatment for omphalitis caused by *T. pyogenes* to prevent multidrug resistance.

## Data availability statement

The raw data supporting the conclusions of this article will be made available by the authors, without undue reservation.

## Ethics statement

The animal study was approved by this study was approved by the Institutional Animal Care and Use Committee (IACUC) at Kyungpook National University (No. KNU-2023-0280). The study was conducted in accordance with the local legislation and institutional requirements.

## Author contributions

YK: Conceptualization, Formal analysis, Investigation, Methodology, Visualization, Writing – original draft. MJJ: Data curation, Formal analysis, Investigation, Validation, Visualization, Writing – original draft. JP: Validation, Resources, Writing – review & editing. KSC: Conceptualization, Formal analysis, Funding acquisition, Supervision, Writing – original draft, Writing – review & editing.

## Footnotes

^1^ http://www.ncbi.nlm.nih.gov

^2^ http://www.geneious.com
