# Supplementary material for: Case report: Omphalitis caused by Trueperella pyogenes infection in a Korean indigenous calf
Source: Front Vet Sci. 2024 May 30;11:1362352. doi: 10.3389/fvets.2024.1362352 (PMC11169830; doi:10.3389/fvets.2024.1362352)
Supplement: Supplementary file 1 [file Table_1.PDF]

Supplementary Table 1. Primer information used for amplification of virulence factors of *Trueperella pyogenes*

Target genes	Sequences (5′−3′)	Sizes (bp)
cbpA	GCAGGGTTGGTGAAAGAGTTTACT	124
	GCTTGATATAACCTTCAGAATTTGCA	
fimC	TGTCGAAGGTGACGTTCTTCG	843
	CAAGGTCACCGAGACTGCTGG	
fimA	CACTACGCTCACCATTCACAAG	605
	GCTGTAATCCGCTTTGTCTGTG	
fimG	ACGCTTCAGAAGGTCACCAGG	929
	ATCTTGATCTGCCCCCATGCG	
nanP	TTGAGCGTACGCAGCTCTTC	150
	CCACGAAATCGGCCTTATTG	
nanH	CGCTAGTGCTGTAGCGTTGTTAAGT	781
	CCGAGGAGTTTTGACTGACTTTGT	
plo	CAGTCAAGGTGAGTGAGTGGAAA	773
	CTTGAACTGGGAAA	

cbpA: collagen-binding protein; fim: fimbriae; nan: neuraminidase; plo: pyolysin

Supplementary Table 2. Antibiotic sensitivity pattern of *Trueperella pyogenes* isolated in this study

Antimicrobial agent	Disk content (μg)	Diameter zone (mm)		Result
		R (resistant)	S (sensitive)	
Amoxicillin	10	≤16	≥17	27
Penicillin G	10 IU	≤19	≥24	–
Ceftiofur	30	≤17	≥21	30
Oxytetracycline	30	≤14	≥19	–
Streptomycin	10	≤11	≥15	10
Erythromycin	15	≤15	≥21	19
Ciprofloxacin	5	≤15	≥21	37
Enrofloxacin	5	≤16	≥23	37
Ofloxacin	5	≤12	≥16	35
Florfenicol	30	≤18	≥22	30
Clindamycin	2	≤15	≥19	–
Lincomycin	2	≤9	≥15	–

'–': no inhibition zone
