# Supplementary material for: Case report: Omphalitis caused by Trueperella pyogenes infection in a Korean indigenous calf
Source: Front Vet Sci. 2024 May 30;11:1362352. doi: 10.3389/fvets.2024.1362352 (PMC11169830; doi:10.3389/fvets.2024.1362352)

Supplementary Figure 1. Dot plots of white blood cell population created using ProCyte Dx hematology analyzer. The number of neutrophils was increased in this calf with omphalitis (A) compared to normal calf (B).

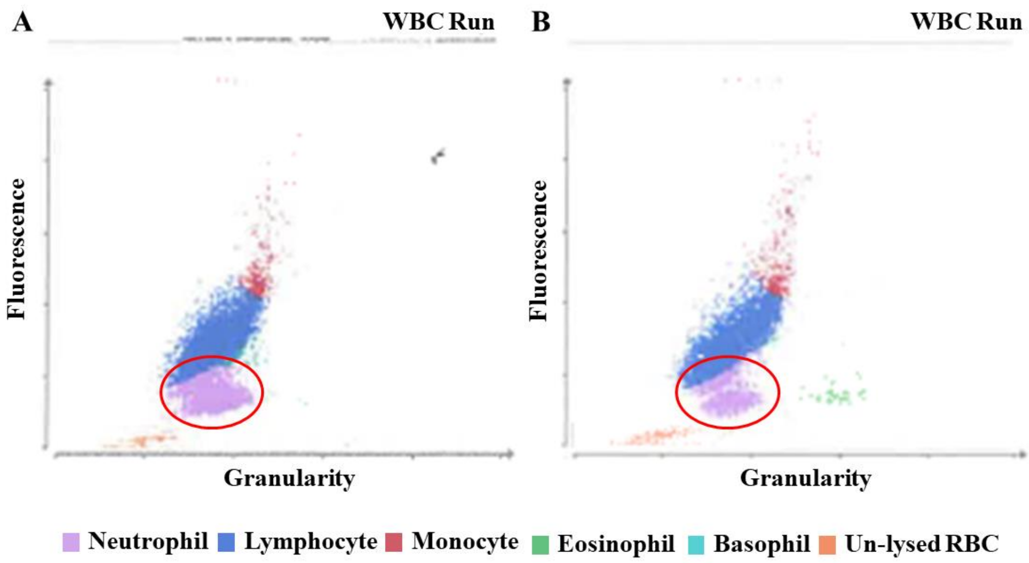

Supplementary Figure 2. Phylogenetic analysis of *Trueperella pyogenes* based on the *plo* gene partial sequences (773 bp) identified in this calf.

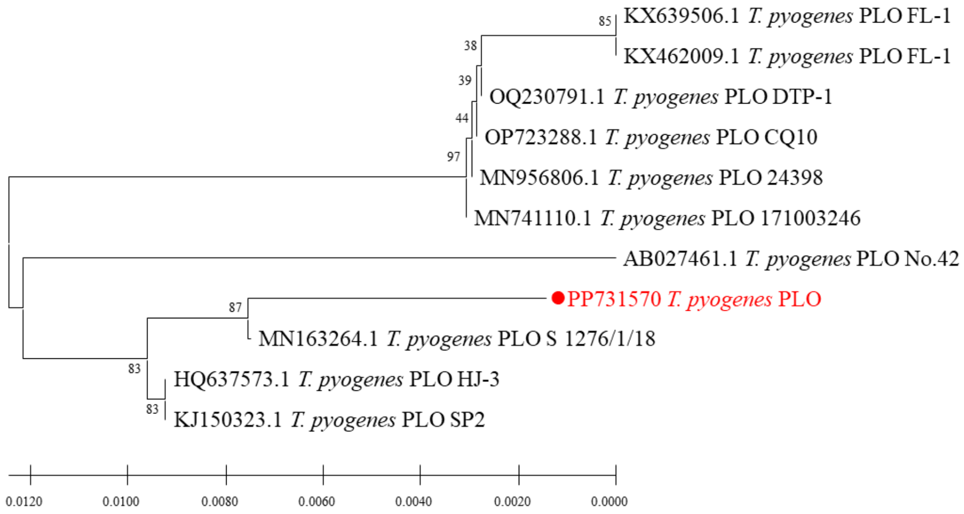

Supplement: Supplementary file 2 [file Image_1.PDF]
